# Supplementary material for: Organization of olfactory centres in the malaria mosquito Anopheles gambiae
Source: Nat Commun. 2016 Oct 3;7:13010. doi: 10.1038/ncomms13010 (PMC5063964; doi:10.1038/ncomms13010)
Supplement: Supplementary Information — Supplementary Figures 1-7, Supplementary Tables 1-5 and Supplementary References [file ncomms13010-s1.pdf]

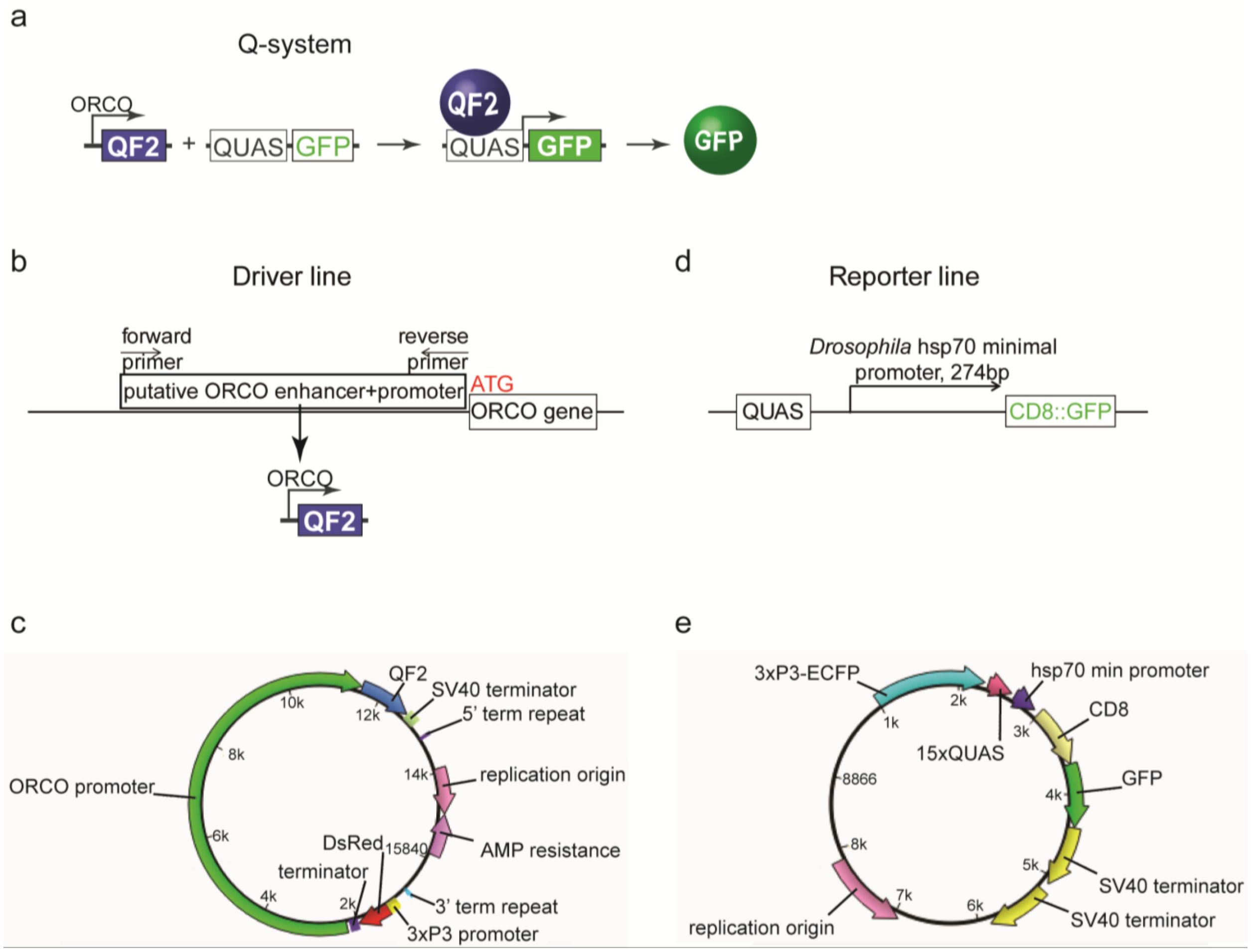

Supplementary Figure 1. DNA constructs for *Anopheles gambiae* transgenesis

a. The binary Q-system for transgenic expression consists of driver (*Orco-QF2*) and reporter (*QUAS-GFP*) components that together result in GFP expression in cells with Orco promoter expression. Binary expression systems can amplify expression from weak promoters.

b, c. Construction of the *Orco-QF2* driver plasmid. The predicted 9312 bp Orco enhancer+promoter region was PCR amplified from genomic DNA and cloned upstream of QF2 in a piggyBac vector containing a 3xP3-DsRed marker.

d, e. Construction of the *QUAS-mCD8:GFP* reporter plasmid. Fifteen copies of the QF-binding sequence (*15xQUAS*) were placed upstream of the *Drosophila* minimal hsp70 TATA promoter in a piggyBac vector containing the 3xP3-CFP marker. A CD8:GFP-SV40 cassette from *Drosophila QUAS-mCD8GFP*[1] was cloned into this vector.

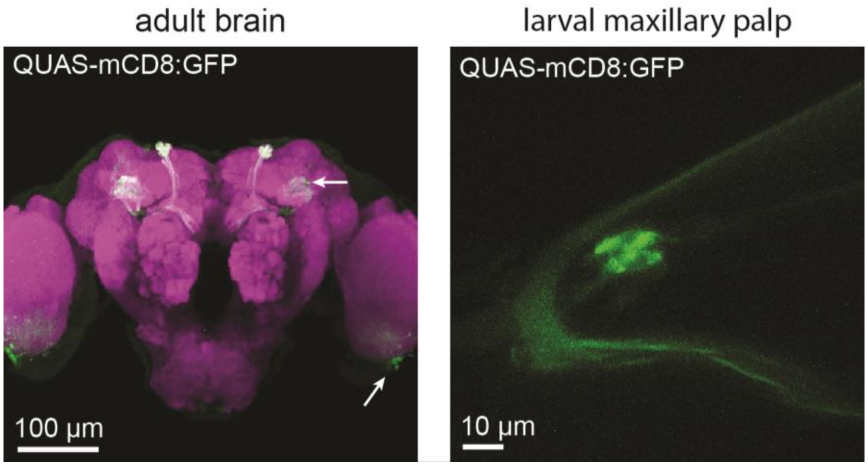

Supplementary FIgure 2. Background expression pattern of *QUAS-CD8:GFP* transgenic animals*.*

Shown is a flattened confocal z-stack demonstrating weak driver-independent expression of the *QUAS-CD8:GFP* reporter in mushroom body and optic lobe neurons (arrows) in the adult brain, as well as weakly in 3 cells in the larval maxillary palp. Scale bars: left, 100 µm; right, 10 µm.

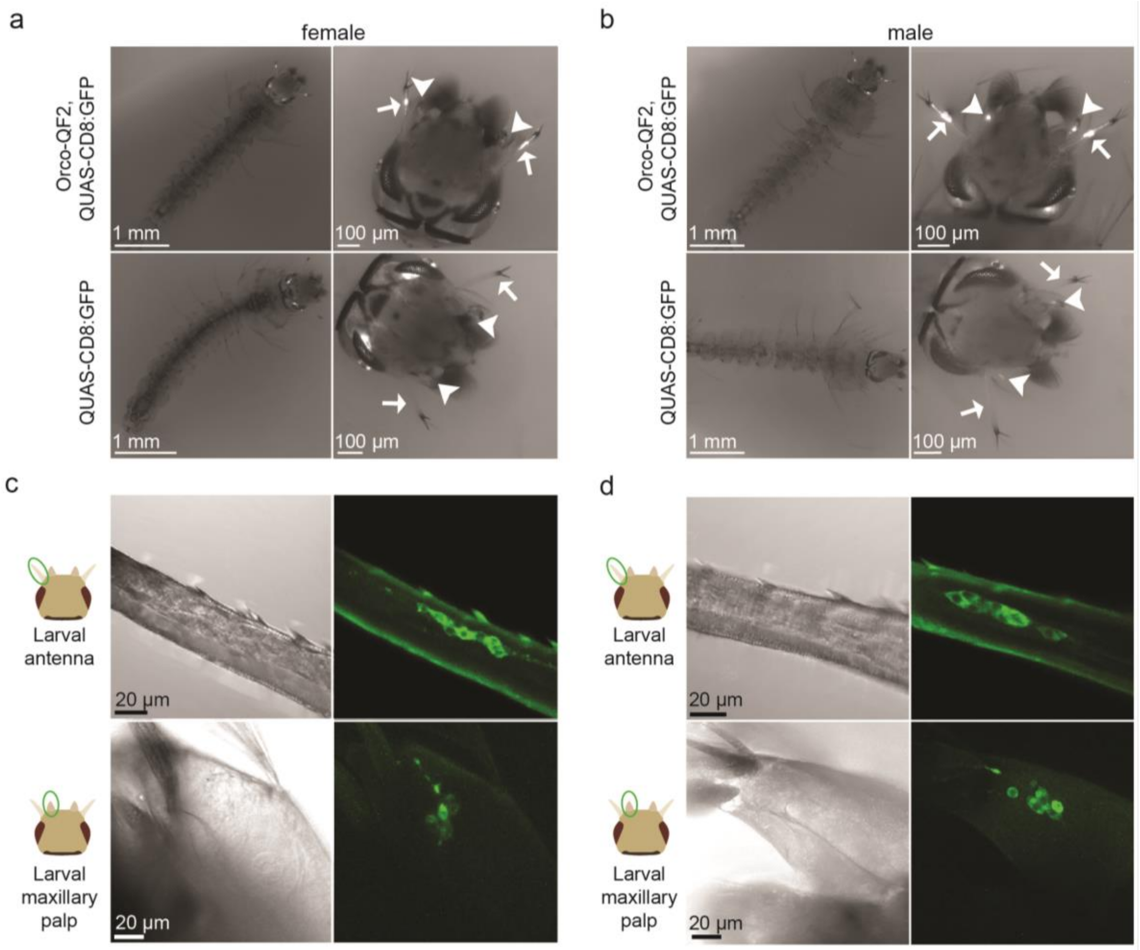

Supplementary Figure 3. ***Orco-QF2*** **expression in olfactory neurons in larva.**

a-b. Membrane targeted GFP expression in third or fourth instar female (a, top rows) and male (b, top rows) larvae. Bottom row shows images of female (a, bottom rows) and male (b, bottom rows) *QUAS-mCD8:GFP* controls. GFP expression is visible in antennae (arrows) and maxillary palps (arrowheads) of transgenic animals (top row). GFP expression is not found in *QUAS-mCD8:GFP* control antennae (bottom row, arrows), but is weakly found in *QUAS-mCD8:GFP* control maxillary palps (bottom row, arrowheads). Transgenic animals also express fluorescent DsRed and CFP markers in the eye, which results in residual fluorescence in the GFP channel. Genotype: *Orco-QF2*, *QUAS-mCD8:GFP.* Scale bars: a and b, left panels: 1 mm; a and b, right panels: 100 µm.

c-d. High-magnification confocal images of GFP expression in the antennae (c and d, top rows) and maxillary palps (c and d, bottom rows) of female (c) and male (d) third or fourth instar larvae. Images were acquired in DIC (grey) and GFP (green) channels, which are shown separately for clarity. Scale bars: 20 µm.

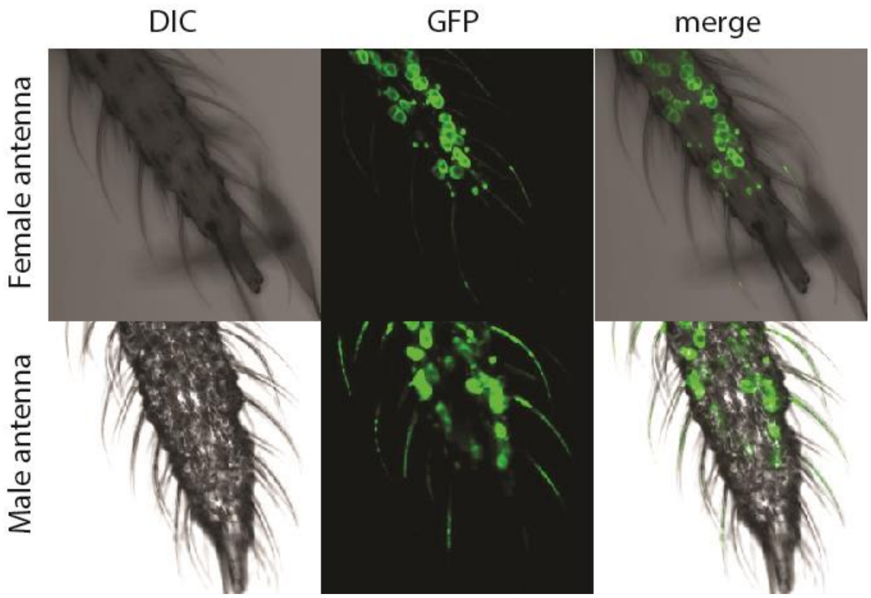

Supplementary Figure 4. GFP fluorescence in adult olfactory tissues.

Shown are differential interference contrast (DIC) microscopy images of un-fixed antennal tissues. Genotype: *Orco-QF2, QUAS-mCD8:GFP*.

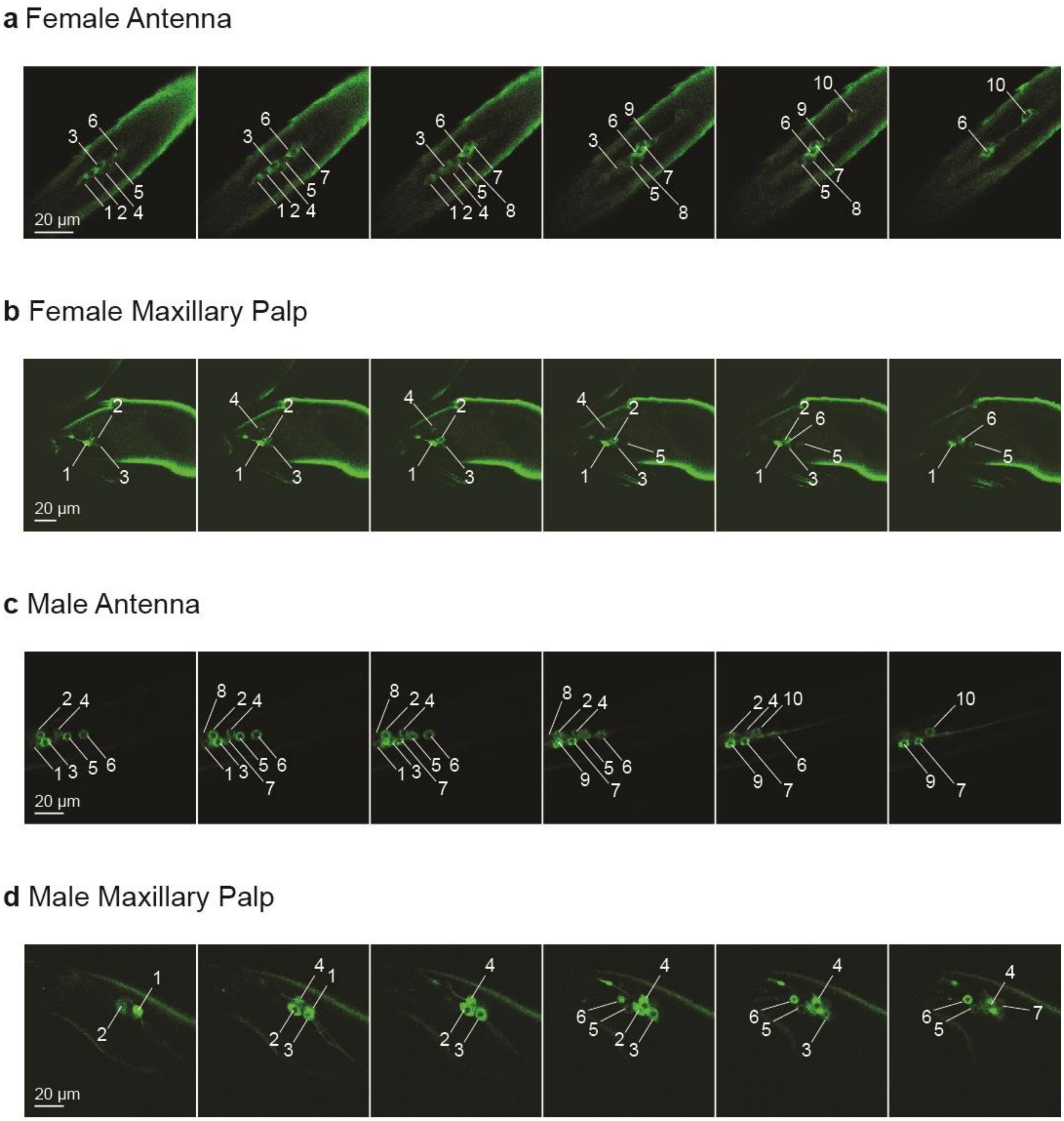

Supplementary Figure 5. Quantification of larval antenna and maxillary palp neurons expressing *Orco-QF2.*

Successive confocal sections showing *Orco-QF2*, *QUAS-CD8:GFP* expression in female antenna (**a**), female maxillary palp (**b**), male antenna (**c**) and male maxillary palp (**d**). Numbers are labels assigned to individual neurons. Scale bars, 20 µm.

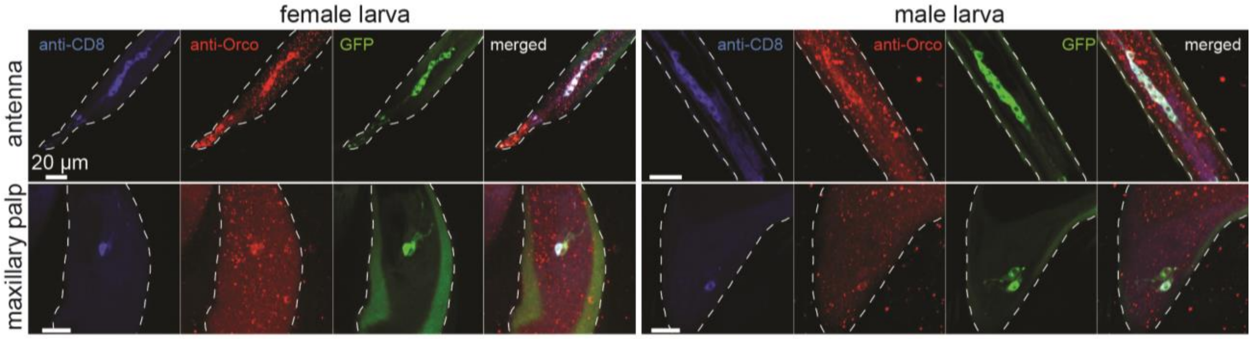

Supplementary Figure 6. *Orco-QF2* drives GFP reporter expression in larval Orco+ neurons.

Antennae (top row) and maxillary palps (bottom row) of third or fourth instar female (left) and male (right) larvae were immunostained with anti-CD8 (blue) and anti-DmOrco (red) antibodies. Images show immunofluorescence of CD8 and Orco, as well as genetically driven GFP (green). Same cells bodies are labelled in all three channels. White dashed line marks borders of the imaged tissue. All images were acquired at the same magnification. Scale bars, 20 µm. Genotype: *Orco-QF2, QUAS-mCD8:GFP.*

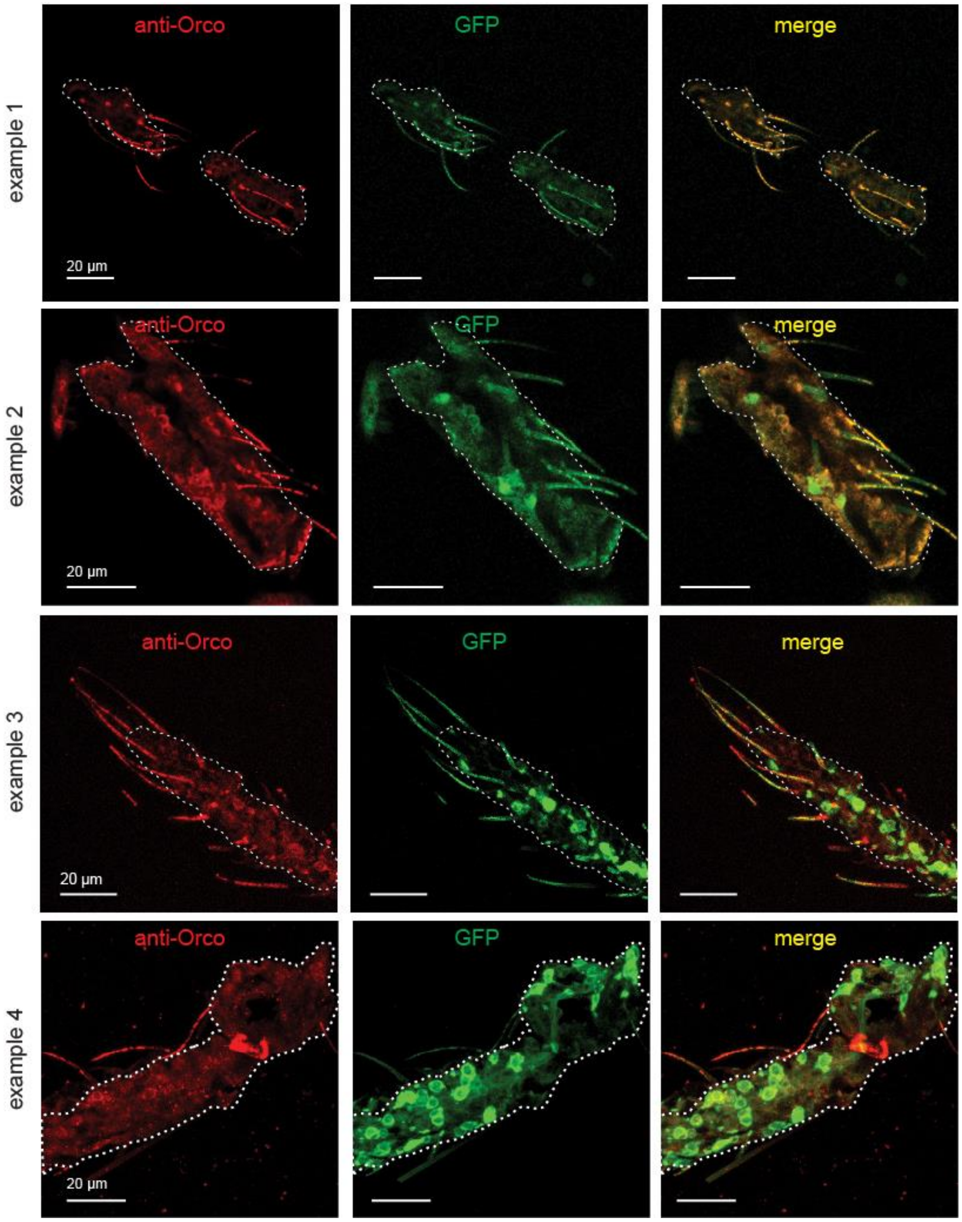

Supplementary Figure 7. Sections of adult female antenna show co-localization between GFP reporter expression and anti-Orco staining.

Four exemplar antennal sections are shown. Sections of adult female antennae were immunostained with anti-DmOrco (red). GFP expression (green) was driven by *Orco-QF2, QUAS-mCD8:GFP*. Co-expression is most notable in the sensilla that contain sensory dendrites. White dashed line outlines borders of the imaged tissue. Scale bars, 20 µm.

Supplementary Table 1. Counts of GFP+ neurons in head appendages of *Orco-QF2, QUAS-CD8:GFP* larvae.

Tissue	# of GFP+ cells
**Female Antennae**	
f_antenna1	10
f_antenna2	9
f_antenna3	9
f_antenna4	11
f_antenna5	11
f_antenna6	9
f_antenna7	9
f_antenna8	9
f_antenna9	10
f_antenna10	10
f_antenna11	10
f_antenna12	10
f_antenna13	8
f_antenna14	11
f_antenna15	10
f_antenna16	11
f_antenna17	12
f_antenna18	9
f_antenna19	8
f_antenna20	11
**median**	**10**
**interquartile range**	**2**
**n**	**20**
**Female Maxillary Palps**	
f_palp1	6
f_palp2	5
f_palp3	5
f_palp4	5
f_palp5	5
f_palp6	6
f_palp7	7
f_palp8	6
f_palp9	6
f_palp10	5
f_palp11	6
f_palp12	6
f_palp13	5
f_palp14	6

f_palp15	5
f_palp16	4
f_palp17	5
f_palp18	5
f_palp19	4
f_palp20	5
**median**	**5**
**interquartile range**	**1**
**n**	**20**
**Male Antennae**	
m_antenna1	9
m_antenna2	9
m_antenna3	10
m_antenna4	11
m_antenna5	12
m_antenna6	9
m_antenna7	11
m_antenna8	12
m_antenna9	11
m_antenna10	11
m_antenna11	11
m_antenna12	11
m_antenna13	9
m_antenna14	14
**median**	**11**
**interquartile range**	**1.75**
**n**	**14**
**Male Maxillary Palps**	
m_palp1	4
m_palp2	6
m_palp3	5
m_palp4	6
m_palp5	5
m_palp6	6
m_palp7	6
m_palp8	4
m_palp9	5
m_palp10	7
m_palp11	6
m_palp12	5
m_palp13	5
m_palp14	6
m_palp15	5

m_palp16	5
m_palp17	5
m_palp18	5
m_palp19	6
m_palp20	6
**median**	**5**
**interquartile range**	**1**
**n**	**20**

Supplementary Table 2. Quantification of anti-ORCO and anti-CD8 staining in larval appendages.

Tissue	# of CD8+ cells	# of Orco+ cells	# of GFP+ cells		Proportion of Orco cells that are CD8+	Proportion of CD8 cells that are Orco+
**Female antennae**						
f_antenna1	9	9	9		1.0	1.00
f_antenna2	9	8	9		1.0	0.89
f_antenna3	8	7	8		1.0	0.88
f_antenna4	10	10	10		1.0	1.00
f_antenna5	8	8	8		1.0	1.00
f_antenna6	8	8	8		1.0	1.00
f_antenna7	11	11	11		1.0	1.00
f_antenna8	11	11	11		1.0	1.00
median	***9.00***	***8.50***	***9.00***	mean	***1.00***	***0.97***
interquartile range	***2.25***	***2.25***	***2.25***	stderr	***0.00***	***0.02***
**Female maxillary palps**						
f_palp1	2	2	5		1.0	1.00
f_palp2	2	2	5		1.0	1.00
f_palp3	1	2	5		0.5	1.00
f_palp4	1	1	5		1.0	1.00
f_palp5	1	1	5		1.0	1.00
f_palp6	3	3	7		0.7	0.67
f_palp7	2	2	6		1.0	1.00
f_palp8	4	3	7		1.0	0.75
f_palp9	2	2	5		1.0	1.00
median	***2.00***	***2.00***	***5.00***	mean	***0.91***	***0.94***
interquartile range	***1.00***	***0.00***	***1.00***	stderr	***0.06***	***0.04***
**Male antennae**						
m_antenna1	9	9	12		1.0	1.00
m_antenna2	9	9	9		1.0	1.00
m_antenna3	10	10	10		1.0	1.00
m_antenna4	12	12	12		1.0	1.00
m_antenna5	10	10	12		1.0	1.00
m_antenna6	10	10	10		1.0	1.00
m_antenna7	9	8	10		1.0	0.89
m_antenna8	12	12	12		1.0	1.00
median	***10.00***	***10.00***	***11.00***	mean	***1.00***	***0.99***
interquartile range	***1.50***	***1.50***	***2.00***	stderr	***0.00***	***0.01***
**Male maxillary palps**						

m_palp1	2	3	7		0.67	1.00
m_palp2	2	2	6		1.00	1.00
m_palp3	2	2	5		1.00	1.00
m_palp4	1	1	6		1.00	1.00
m_palp5	2	2	6		1.00	1.00
m_palp6	2	2	7		1.00	1.00
m_palp7	1	1	5		1.00	1.00
m_palp8	2	2	6		1.00	1.00
m_palp9	2	2	6		1.00	1.00
median	***2.00***	***2.00***	***6.00***	mean	***0.96***	***1.00***
interquartile range	***0.00***	***0.00***	***0.00***	stderr	***0.04***	***0.00***

Supplementary Table 3. Quantification of anti-ORCO and anti-CD8 staining in adult appendages.

Tissue	# of CD8+ cells	# of Orco+ cells		Proportion of Orco cells that are CD8+	Proportion of CD8 cells that are Orco+
**Female antennae (partial)**					
f_antenna1	75	71		1.0	0.95
f_antenna2	54	51		1.0	0.94
f_antenna3	61	57		1.0	0.93
f_antenna4	64	59		1.0	0.92
f_antenna5	37	36		1.0	0.97
f_antenna6	35	34		1.0	0.97
			mean	***1.00***	***0.95***
			stderr	***0.00***	***0.01***
**Female maxillary palps (partial)**					
f_palp1	76	72		1.0	0.95
f_palp2	79	77		1.0	0.97
f_palp3	70	67		1.0	0.96
f_palp4	157	149		1.0	0.95
f_palp5	47	47		1.0	1.00
			mean	***1.00***	***0.97***
			stderr	***0.00***	***0.01***
**Female proboscis (partial)**					
f_proboscis1	31	28		1.0	0.90
f_proboscis2	38	28		1.0	0.74
f_proboscis3	21	18		1.0	0.86
f_proboscis4	18	15		1.0	0.83
f_proboscis5	17	15		1.0	0.88
			mean	***1.00***	***0.84***
			stderr	***0.00***	***0.03***
**Male antennae (partial)**					
m_antenna1	65	62		1.0	0.95
m_antenna2	70	63		1.0	0.90
m_antenna3	43	36		1.0	0.84
m_antenna4	40	37		1.0	0.93
m_antenna5	32	30		1.0	0.94
			mean	***1.00***	***0.91***

			stderr	***0.00***	***0.02***
**Male maxillary palps (partial)**					
m_palp1	19	20		0.95	1.00
m_palp2	32	28		1.00	0.88
m_palp3	42	42		1.00	1.00
m_palp4	33	30		1.00	0.91
m_palp5	14	14		1.00	1.00
			mean	***0.99***	***0.96***
			stderr	***0.01***	***0.03***
**Male proboscis (partial)**					
m_proboscis1	34	33		1.0	0.97
m_proboscis2	36	31		1.0	0.86
			mean	***1.00***	***0.92***
			stderr	***0.00***	***0.05***

Supplementary Table 4. Quantification of glomerular innervation patterns in the mosquito antennal lobes.

Gender, manipulation	Total #	# of Orco+	# of backfilled	# of Orco+ and backfilled	Note
**Female, antennal backfill**					
f_ant_AL1	68	32	61	30	
f_ant_AL2	67	34	57	32	
f_ant_AL3	70	37	64	35	
**Female, maxillary palp backfill**					
f_mp_AL1	67	30	5	2	
f_mp_AL2	68	33	5	2	This AL is shown in Fig 4A
f_mp_AL3	67	36	5	2	
f_mp_AL4	67	35	5	2	
**Male, antennal backfill**					
m_ant_AL1	67	34	59	32	
m_ant_AL2	68	36	61	34	
**Male, maxillary palp backfill**					
m_mp_AL1	67	33	5	2	This AL is shown in Fig 4A

Supplementary Table 5. Summary of antennal lobe innervation patterns and new nomenclature.

This study				Ghaninia et al, 2007
ORCO>GFP	ANT+	MP+	Glomeruli nomenclature	Glomeruli nomenclature
■	■		1	VM2
■	■		2	JO complex
	■		3	AV1
■	■		4	AV2
■	■		5	JO complex
■	■		6	JO complex
■	■		7	JO complex
■	■		8	AM2
■	■		9	AM4
■	■		10	AD1
■	■		11	JO complex
	■		12	VM3
■	■		13	AM1
■	■		14	JO complex
■	■		15	JO complex
■	■		16	DM1
■	■		17	DM3
	■		18	JO complex
	■		19	AL1
■	■		20	AL2
■	■		21	PV1
■	■		22	AM5
■	■		23	AD2
■	■		24	VM7
	■		25	AM3
■	■		26	PV5
	■		27	AL3
■	■		28	DM5
	■		29	AL4
■	■		30	AL5
	■		31	PV4
	■		32	no correspondence
	■		33	VM9
	■		34	no correspondence
■	■		35	PV2
■	■		36	VM6
■	■		37	JO complex

			38	AD4
			39	AL6
			40	DM4
			41	DM6
			42	DM2
			43	no correspondence
			44	no correspondence
			45	DM8
			46	DM9
			47	no correspondence
			48	VM4
			49	no correspondence
			50	no correspondence
			51	AD5
			52	AD7
			53	DC1
			54	DC2
			55	DM7
			56	DC5
			57	no correspondence
			58	no correspondence
			59	no correspondence
			60	no correspondence
			61	no correspondence
			62	DM12
			63	DM11
			64	DM10
			65	no correspondence
			66	AD10
			67	DC8
			68	DC7

Supplementary Reference

1 Potter, C. J., Tasic, B., Russler, E. V., Liang, L. & Luo, L. The Q system: a repressible binary system for transgene expression, lineage tracing, and mosaic analysis. *Cell* **141**, 536-548 (2010).
